# Supplementary material for: Development of Wash-Durable Antimicrobial Cotton Fabrics by In Situ Green Synthesis of Silver Nanoparticles and Investigation of Their Antimicrobial Efficacy against Drug-Resistant Bacteria
Source: Antibiotics (Basel). 2022 Jun 27;11(7):864. doi: 10.3390/antibiotics11070864 (PMC9311951; doi:10.3390/antibiotics11070864)
Supplement: Supplementary file 1 [file antibiotics-11-00864-s001.zip › antibiotics-1748613-supplementary.pdf]

Supplementary

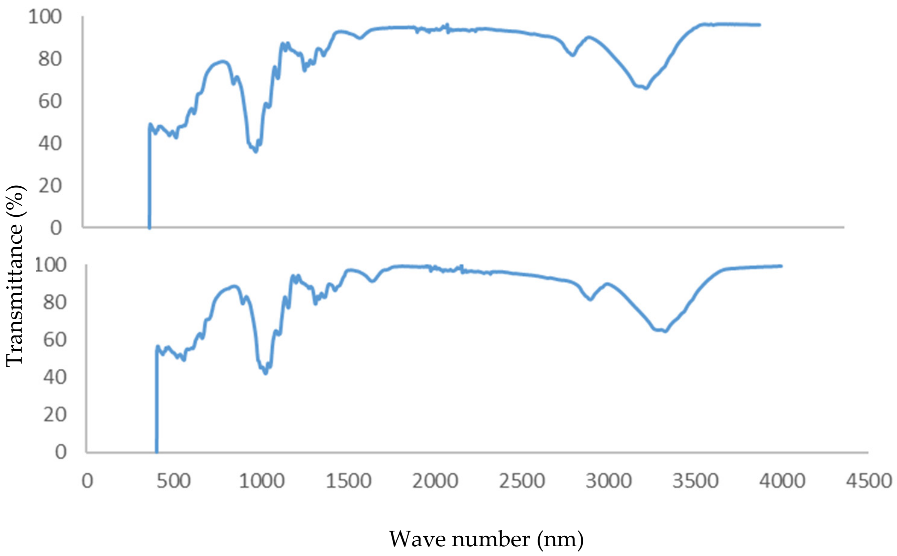

Figure S1. Fourier-Transform Infrared spectra of (A) Mercerized cotton fabric without AgNP treatment (B) AgNP treated mercerized cotton fabric.

Table S1. Zeta-Potential of Mercerized and un-mercerized cotton fabric.

SAMPLE	ZETA POTENTIAL (mV)
Without Mercerization	-35.27
Mercerization	-60.9

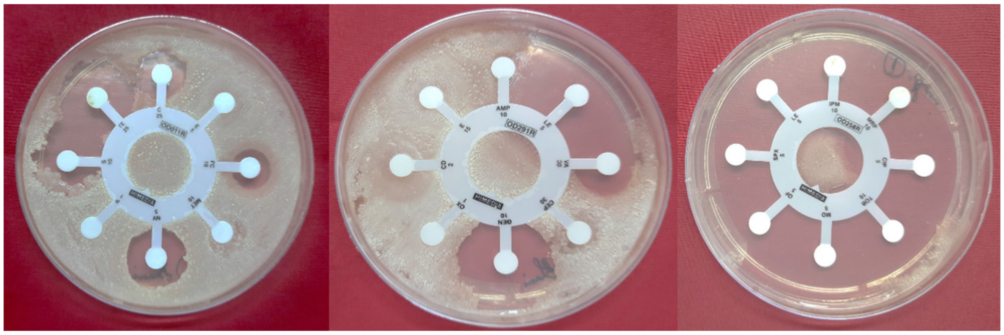

Figure S2: Antibiotic disc diffusion assay against *B. licheniformis*.

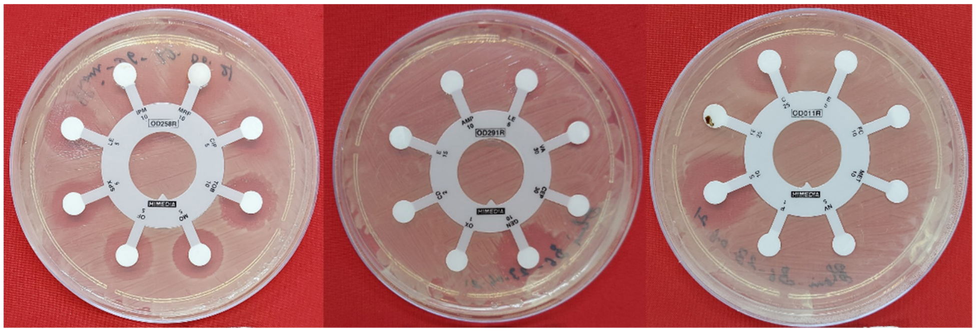

Figure S3: Antibiotic disc diffusion assay against *E. coli*.

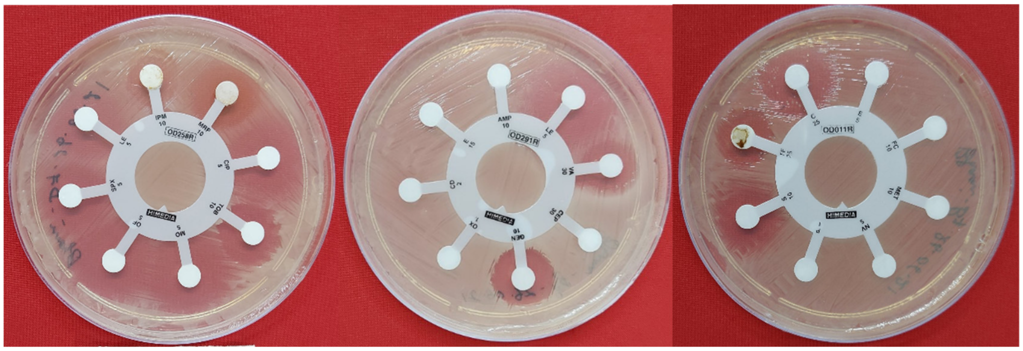

Figure S4. Antibiotic disc diffusion assay against *K. pneumoniae*.

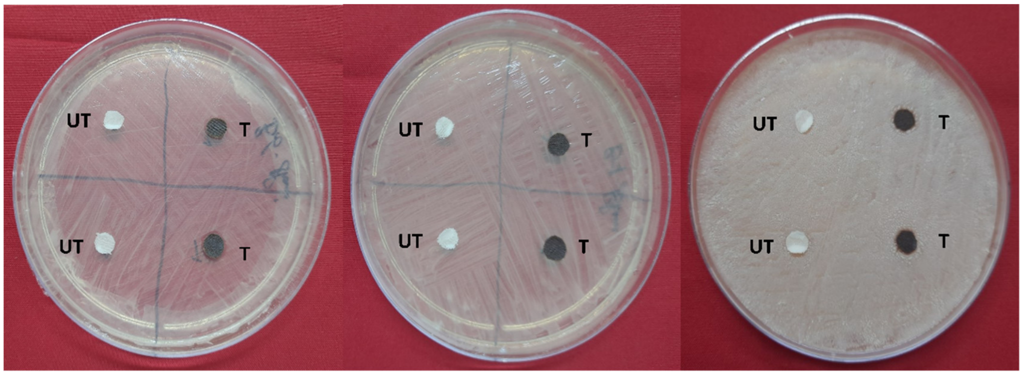

E. coli *K. pneumoniae* *B. licheniformis*

Figure S5: Disc diffusion assay of the fabric (control and AgNP treated) against the three test bacteria.

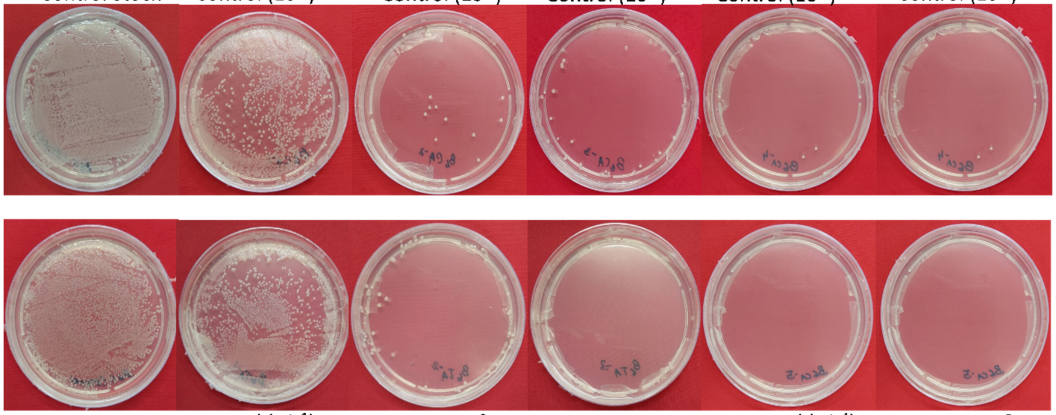

Figure S6. The number of *E. coli* colonies formed (CFU mL^{-1}) were counted after exposing to both treated and untreated fabric.

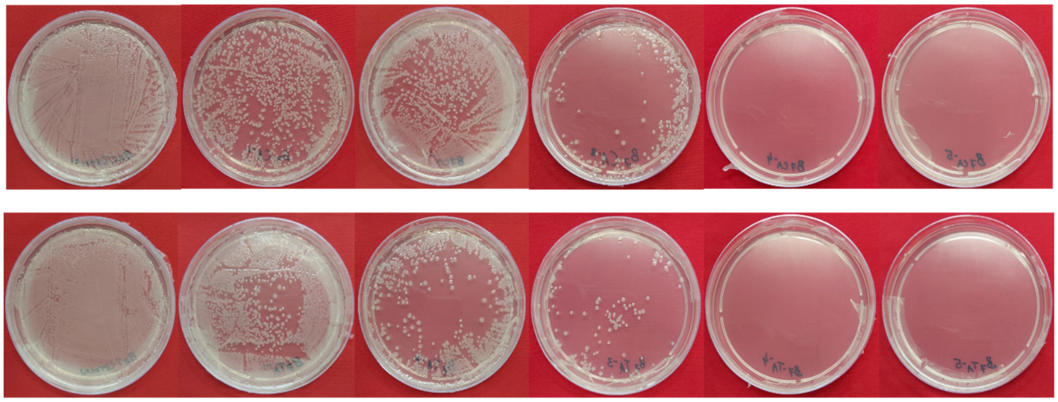

Figure S7. The number of *K. pneumoniae* colonies formed (CFU mL^{-1}) were counted after exposing to both treated and untreated fabric.

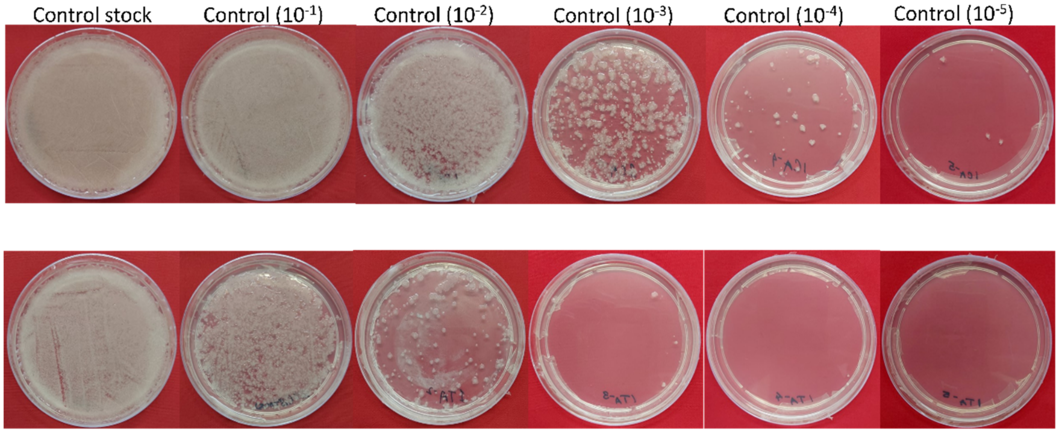

Figure S8. The number of *B. licheniformis* colonies formed (CFU mL^{-1}) were counted after exposing to both treated and untreated fabric.
